# Identification of New Human P2X7 Antagonists Using Ligand- and Structure-Based Virtual Screening

**DOI:** 10.1021/acs.jcim.5c00552

**Published:** 2025-06-26

**Authors:** Marika Zuanon, Andrea Brancale, Mark T. Young

**Affiliations:** † School of Biosciences, 2112 Cardiff University, Sir Martin Evans Building, Cardiff CF10 3AT, United Kingdom; ‡ Department of Organic Chemistry, 52735 University of Chemistry and Technology, Prague 166 28, Czech Republic

## Abstract

P2X7 receptors, a subtype of ATP-gated cation channel, have gained attention due to their involvement in inflammatory and neurodegenerative diseases, chronic pain, and cancer. However, despite extensive medicinal chemistry efforts, no P2X7 antagonists have reached clinical approval due to suboptimal pharmacokinetic properties, poor selectivity, and insufficient efficacy in comparison to placebo controls. To address these challenges, we employed a virtual screening workflow integrating ligand-based and structure-based approaches to identify novel P2X7 allosteric antagonists. A 3D pharmacophore model derived from three known P2X7 antagonists (A740003, A804598, and JNJ47965567) was used to filter four libraries of commercially available compounds (approximately 10,000,000 total). These compounds were docked into a human P2X7 homology model and ranked by four distinct scoring functions. Eleven compounds were selected based on drug-like properties and key interactions with residues lining the target pocket. Among those, six compounds inhibited P2X7 activation in a YO-PRO 1 dye uptake assay (30 μM), while just two of those (**2** and **9**) were also active in a Membrane Potential Red assay (10 μM). Further screening of 10 analogues of **2** and **9** led to the identification of **2g**, which displayed comparable potency (IC_50_ = 1.31 μM) to **2** (IC_50_ = 1.88 μM) in the YO-PRO 1 dye uptake assay. Docking studies of **2g** within the negative allosteric pocket provided insights into its binding mode and key interacting residues. These findings offer a promising starting point for the development of optimized P2X7 antagonists.

## Graphical abstract

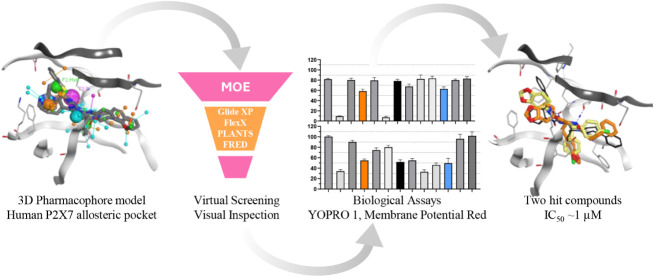

## Introduction

The P2X7 receptor belongs to the P2X purinergic family (P2X1–7) of trimeric nonselective cation channels gated by ATP.^1^ Among the P2X receptors, P2X7 is activated by high concentrations of extracellular ATP (∼100 μM), which are usually present in situations of cell death, such as infection and inflammation, where it functions as a “death receptor”.^2–4^

The functions of P2X7 are related to its channel properties and also to its extended cytoplasmic domain. The opening of the P2X7 ion channel leads to intracellular ion redistribution, causing K^+^ depletion and Ca^2+^ influx,^5–7^ which promote the release of proinflammatory cytokines via the assembly and activation of the NLRP3 inflammasome and apoptosis through cytochrome c release, respectively.^8^

Furthermore, the extended cytoplasmic domain is associated with protein interactions, coupling with intracellular signaling,^9^ and it is also thought to be responsible for the opening of a nonselective membrane “macropore” upon prolonged P2X7 activation.^2^ The macropore allows high molecular weight molecules (up to 900 Da) to permeate the membrane,^10,11^ and it is critical for cell death mechanisms, including pyroptosis and apoptosis.^5–7^ The macropore property associated with cell death is absent in both the splice isoform P2X7B, which lacks the C-terminal cytoplasmic domain^12^ and the nonfunctional variant nfP2X7.^13^ These two receptors are mainly expressed by cancer cells, where they appear to promote cell proliferation.^14^

Given its proinflammatory and cytotoxic functions, P2X7 has raised interest as a therapeutic target for the treatment of cancer, neurodegenerative, inflammatory, and infectious diseases.^7,15,16^

Several P2X7 antagonists have been discovered by pharmaceutical companies through extensive high-throughput screening (HTS) and medicinal chemistry campaigns^17–19^ (Supporting material Figures 1 and 2). However, no P2X7 antagonist has yet received clinical approval, largely due to a lack of species crossover activity to human P2X7, suboptimal pharmacokinetic profiles, and insufficient efficacy in comparison to placebo controls.^18,20^

To address these challenges, computational approaches, such as virtual screening (VS), have the potential to accelerate the discovery of novel P2X7 modulators by reducing the time and costs associated with traditional HTS.^21^ VS involves virtually docking large libraries of small molecules into the 3D structure of a biological target, followed by scoring their binding conformers.^22^ This strategy allows for the selection of a smaller set of promising compounds for experimental evaluation.

VS techniques rely on two key sources of information: the 3D structure of the biological target (structure-based drug design, SBDD) or the structural characteristics of small molecules known to interact with the target (ligand-based drug design, LBDD).^23^ Structural data for the biological target may come from experimental methods such as X-ray crystallography or cryoelectron microscopy (cryo-EM). In cases where structural information for the target is not available, it is possible to create a homology model using available structures as a template. Conversely, knowledge of the structure of active small molecules can guide the identification of novel compounds with similar chemical features and shape-based properties critical for target binding.^23^ Both SBDD and LBDD can be used to identify new P2X7 ligands (either used separately or in combination) as several structures of this and other homologous P2X receptors have been resolved in recent years,^11,24–26^ together with the discovery of specific small-molecule modulators.^18,20^

Evidence from P2X7 receptor structures and point mutation studies has revealed the presence of at least four ligand-binding pockets (ATP (orthosteric), negative allosteric,^25^ positive allosteric^27^ and GDP/GTP^11^ distributed in the different domains of the P2X7 protein. Among these, the ATP and the negative allosteric pockets (Figure 1A), which are located in the extracellular domain and distinct from each other, have been used for SBDD to date.

**Figure 1:**
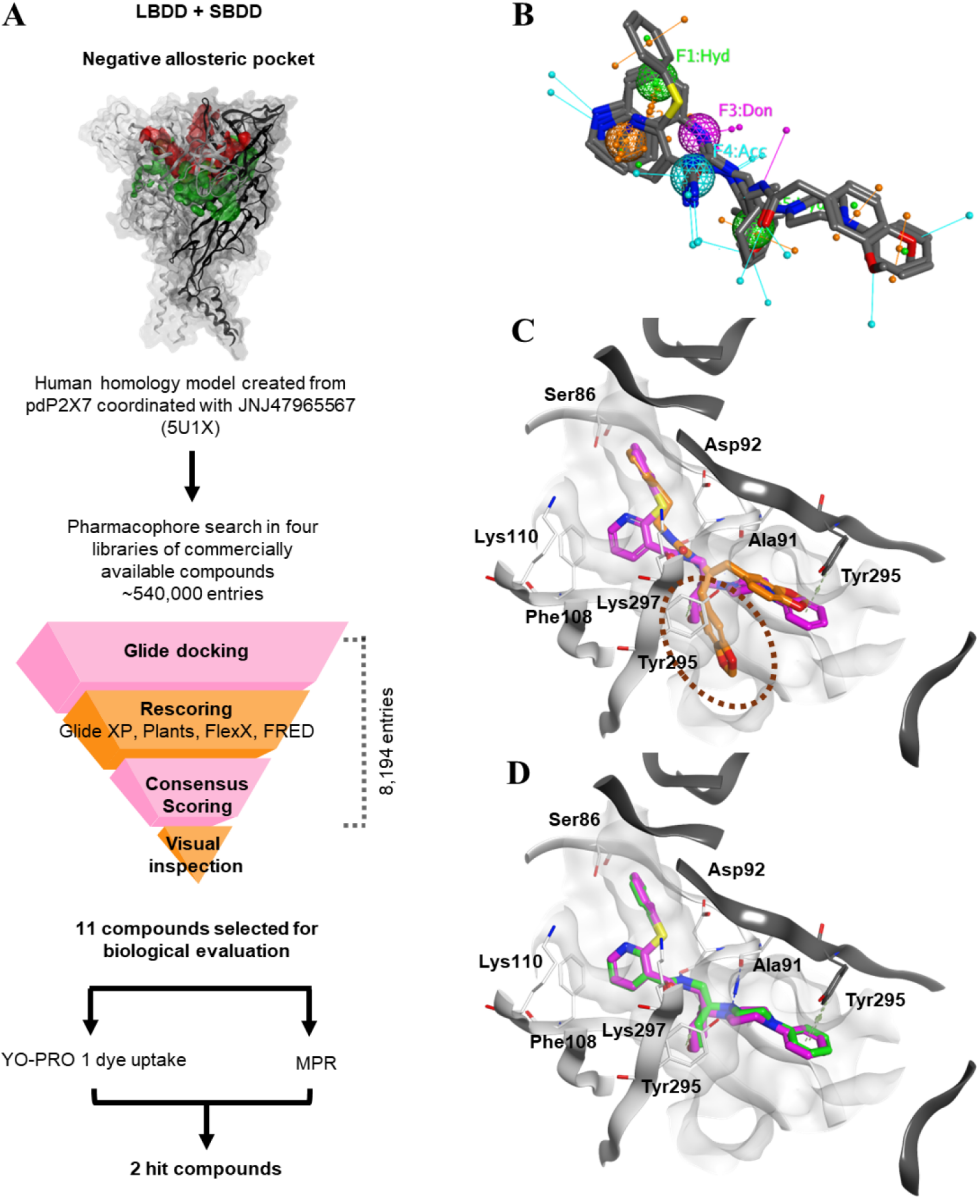
Virtual screening and visual inspection in the negative allosteric pocket. (A) Schematic of the VS performed in the hP2X7 negative allosteric pocket (red surface), which is adjacent to the ATP pocket (green surface). (B) Five features selected for the pharmacophore search superposing A740003 (PDB ID: 5U1U), A804598 (PDB ID: 5U1 V), and JNJ47965567 (PDB ID: 5U1X); (F1 and F5 in green are two lipophilic groups; F2 in orange is an aromatic group; F3 in pink, and F4 in cyan are two hydrogen groups, donor and acceptor, respectively). (C) Visualization of the novel area (highlighted by the oval shape) of the pocket occupied by the entries **2**, **4**, and **7** found in the VS. (D) The JNJ47965567 docked pose in VS (green) overlaps that of the cocrystallized ligand (pink) in pdP2X7 (PDB ID 5U1X). LBDD, ligand-based drug design; MPR, Membrane Potential Red assay; pdP2X7, giant panda P2X7; and SBDD, structure-based drug design.

The three ATP pockets are confined by two adjacent subunits, and when ATP binds, it initiates all the protein conformational changes that lead to the opening of the ion channel^28^ (Figure 1A). This binding site is highly conserved among all the P2X receptors,^29^ making the identification of subtype-selective orthosteric antagonists a challenge.

The orthosteric pocket has been used in two VS, performed by Caseley^30^ and Zhao,^31^ while our group serendipitously found a P2X7 antagonist, GP25, by initially performing a VS in the P2X4 ATP pocket^32^ (Supporting material Figure 1).

The negative allosteric pockets are adjacent but separated from the ATP-binding sites, and they are found in the intersubunit cavity at the top of the extracellular domain^25^ (Figure 1A). Ligands targeting this pocket prevent ATP-induced conformational changes and, consequently, P2X7 activation. While the residues lining this pocket are similar to those in the P2X3 and P2X4 receptors,^33,34^ the structural differences render it an exclusive binding site for P2X7 antagonists.^25^ Most of the known selective P2X7 antagonists bind to this pocket,^25,35–37^ and recently, it has been shown that they bind in three distinct conformations: shallow, deep, and starfish.^26^

A combination of SBDD and LBDD has been used recently to identify natural products binding to the negative allosteric pocket,^38^ although no functional data were presented to confirm their activity.

In this study, we aimed to identify negative allosteric modulators of hP2X7 by integrating the SBDD and LBDD approaches (Figure 1A). We constructed a 3D pharmacophore model based on shared chemical features of three known P2X7 antagonists (A740003, A804598, and JNJ47965567)^25^ (Figure 1B). This model was used to filter four commercially available compound libraries, yielding a database of 540,000 molecules with desirable features. Subsequently, the database was virtually screened against the negative allosteric pocket of a human P2X7 (hP2X7) homology model based on the giant panda P2X7 structure bound to JNJ47965567 (PDB: 5U1X).^25^ Following rescoring and visual inspection, 11 candidate compounds were selected for experimental validation.

The functional activity of the 11 small molecules was assessed by using YO-PRO 1 dye uptake and membrane potential red (MPR) assays. This initial screening identified two hit compounds, **2** and **9**. Further structure–activity relationship (SAR) studies, involving 10 structural analogues of these hits, led to the discovery of **2g**. Although we could only obtain **2g** at >75% purity, it exhibited an IC_50_ comparable to its parent compound **2** in the YO-PRO 1 dye uptake assay (1.31 μM and 1.88 μM, respectively). Molecular docking of **2g** into the negative allosteric pocket provided insights into its binding mode and improved activity against hP2X7.

## Materials and Methods

### Materials

JNJ47965567 was purchased from Tocris Bioscience. ATP was purchased from Merck. Small-molecule test compounds were purchased from ChemDiv and Enamine (for proof of purchase, see **Supporting material**). HPLC analysis of the most active compounds (**Supporting material**; provided by the vendor) indicated that **2** and **9** were >95% purity, and **2g** was >75% purity.

### Homology Modeling

A model of human P2X7 (hP2X7; UniProt ID Q99572) was generated using the homology modeling tool in MOE v2022.02 (ULC, Chemical Computing Group, Molecular Operating Environment (MOE)). The model was built based on the crystal structure of the giant panda P2X7 receptor in complex with JNJ47965567 (PDB ID: 5U1X),^25^ which presents 3.2 Å resolution and 84.5% amino acid identity with hP2X7. To avoid steric clashes with the ligand in the negative allosteric pocket, the cocrystallized ligand JNJ47965567 was retained as a reference during model construction. Among the generated models, the one with the lowest root-mean-square deviation (RMSD) from the template was selected and refined using the Protein Preparation Wizard tool in Maestro (Schrödinger 2023–2).^39^ Ionization states were assigned at pH 7.0 ± 0.2 using Epik, and the structure was energy minimized with the OPLS4 force field (Schrödinger). Model validation was performed by assessing the Ramachandran plot and conducting a detailed visual inspection.

### Pharmacophore Search

A pharmacophore model was created using MOE by superimposing three of the P2X7 allosteric antagonists cocrystallized with the giant panda P2X7 receptor: A740003 (PDB ID: 5U1U), A804598 (PDB ID: 5U1 V), and JNJ47965567 (PDB ID: 5U1X).^25^ The alignment identified five shared features: two hydrophobic groups, one aromatic group, one hydrogen bond donor, and one hydrogen bond acceptor (Figure 1B). These characteristics were used to filter four libraries of approximately 10,000,000 commercially available compounds (ChemDiv, LifeChem, SPECS, and Enamine). Approximately 540,000 entries satisfied the features selected in the pharmacophore model, and stereoisomers were generated using LigPrep (Schrödinger) with ionization states assigned at pH 7.0 ± 0.2 via Epik.^39^

### Virtual Screening and Consensus Scoring

The filtered library was docked into the negative allosteric pocket of the hP2X7 homology model. A docking grid with a < 20 Å radius was created using the GlideGrid tool (Schrödinger)^40^ centered on the cocrystallized ligand JNJ47965567. Docking was performed with Glide in the Standard Precision (SP) mode, generating three poses per ligand. The top 10% of poses, based on docking scores, was reevaluated in Extra Precision (XP) mode.^40^

The database was further assessed using three additional scoring functions: PLANTS, FlexX, and FRED (OpenEye Scientific software). PLANTS^41^ and FlexX^42^ allow for flexible ligand conformations, while FRED^43^ considers rigid conformers and eliminates those that clash with the protein pocket. A consensus scoring approach was applied, selecting ligands that ranked in the top 25% across all scoring functions. Selected ligand poses were visually inspected using MOE, focusing on interaction quality, alignment with the cocrystallized antagonist, structural diversity, and drug-likeness (adherence to Lipinski’s rule of five). All the tested compound structures were checked for possible PAINS using the online software Swiss ADME.^44^

### Cell Culture

Astrocytoma 1321 N1 (established by Dr. Gaia Pasqualetto)^32^ and HEK-293 (kindly gifted by E. Adinolfi’s group, Ferrara University)^12^ both stably transfected with hP2X7, were used to assess the activity of the small molecules selected from the VS. Cells were maintained in DMEM:F12 (PanBiotech) supplemented with 10% FBS, 1% pen-strep, and 150 μg/mL G418 (PanBiotech).

### YO-PRO 1 Dye Uptake Assay

Astrocytoma 1321 N1 cells (2–2.5 × 10^4^ cells/well)^32^ and HEK-293 cells (4–5 × 10^4^ cells/well)^12^ expressing hP2X7 were seeded in poly-l-lysine-coated 96-well plates (Sarstedt) 24 h before the experiment. YO-PRO 1 dye was prepared at 5 μM in extracellular solution with low divalent cations (ECS-LD) buffer (147 mM NaCl, 10 mM HEPES, 13 mM glucose, 0.2 mM CaCl_2_, 2 mM KCl, pH 7.4) and added to cells after washing them with Dulbecco’s phosphate- buffered saline (DPBS) (PanBiotech) and incubated for 20 min at 37 °C. ATP-induced dye uptake, mediated by hP2X7 activation, was monitored using a Clariostar plate reader (excitation: 485 nm; emission: 538 nm) for 30 cycles (75–150 s/cycle), after recording the baseline for five cycles. hP2X7 activation was quantified at cycle 10 by calculating the fluorescence intensity (FI) ratio (Δ­(F_cycle 10_–F _cycle 1_)/cycle time).

### Membrane Potential Red (MPR) Dye Assay

Cells were plated as described for the YO-PRO 1 dye uptake assay. MPR dye (Molecular Devices) was prepared following the manufacturer’s protocol and diluted 1:10 in ECS-LD buffer. After washing with DPBS, cells were incubated with the dye for 30 min at 37 °C. Fluorescence changes (excitation: 525 nm; emission: 560 nm) were recorded using a Clariostar plate reader during ATP-induced membrane depolarization. Baseline fluorescence was recorded for 5 cycles, and ATP was added via an automated injector (10 μL). Membrane depolarization was quantified as the ratio of the maximal fluorescence change over baseline intensity (Δ­(*F*_max_–F_0_)/F_0_)).

### Cytotoxicity Assay (CellTiter Blue)

HEK-293 WT cells were seeded at 4 × 10^4^ cells/well in 96-well plates precoated with poly-l-lysine. After 24 h, the cells were washed with DPBS and treated with test compounds (10 μM) in 2% FBS-containing medium for 24 h. After this, the medium containing the test compounds was replaced with 20% CellTiter-Blue reagent (Promega) in 2% FBS medium. After a 2-h incubation, fluorescence (excitation: 579 nm; emission: 584 nm) was measured using a Clariostar plate reader. Data were normalized to untreated control cells (100%) and wells without cells (0%).

### Statistical Analysis

Data analysis was carried out using GraphPad Prism version 8 for Windows (GraphPad software, San Diego, California, USA). Data are represented as mean ± SEM. The comparison between independent experiments (N) was performed by normalizing each data set to the mean value of the control before data analysis. To analyze the antagonism effect of the tested compounds on agonist-induced responses, data are represented as a percentage of the mean response obtained by the activity of the agonist in the vehicle. Inhibition concentration–response curves were calculated by applying a nonlinear regression (curve fit) with four parameters after normalizing the data to the maximal agonist response in the vehicle control. Cell viability data were expressed as a percentage of the vehicle-treated samples. Statistical analysis between samples and controls was calculated through one-way ANOVA followed by Dunnett’s multiple comparison test, with *P* < 0.02 considered significant.

## Results

### Pharmacophore Model Development and Virtual Screening Identify 11 Candidate Compounds for Biological Evaluation

To build a pharmacophore model, five cocrystallized ligands bound to the giant panda P2X7 receptor^25^ were analyzed. Using MOE’s Pharmacophore Query Editor, three ligands-A740003 (PDB ID: 5U1U), A804598 (PDB ID: 5U1 V), and JNJ47965567 (PDB ID: 5U1X)-were identified to share the highest number of conserved chemical features. The pharmacophore model was built by selecting two lipophilic groups (F1 in proximity to PHE88; F5 in proximity to TYR295), one aromatic group (F2 in proximity to PHE108 and ILE310), and two hydrogen groups (F3 in proximity to LYS297; F4 in proximity to ASP92) (Figure 1B), and it was used to filter the ligands of four libraries of commercially available compounds (Enamine, ChemDiv, LifeChem, SPECS).

The pharmacophore search filtered 540,643 compounds from an initial pool of 10,000,000 compounds. This subset was processed by LigPrep (Schrödinger), which generated stereoisomers and assigned ionization stated at pH 7 ± 0.2, resulting in a final library of 1,656,322 entries to use for VS.

The library of compounds was screened into the negative allosteric pocket using a Glide workflow. Each compound was docked by Glide Standard Precision (SP), generating three poses per structure, with the top 10% retained for further rescoring using Glide Extra Precision (XP). Three additional scoring methods, PLANTS, FlexX, and FRED—were applied to the selected poses. Consensus scoring, which combined the results from all four scoring functions, led to the identification of 8,194 compounds for visual inspection (Figure 1A).

To guide the inspection process, the interactions established by the five cocrystallized ligands in the giant panda P2X7 (pdP2X7) structure were analyzed. Key residues included ASP92 and LYS297, which frequently formed hydrogen bonds, and LYS110, which was involved in either hydrogen or arene-hydrogen bonding. Notably, AZ10606120 and GW791343 were the only cocrystallized ligands interacting with residues at the pocket’s entrance, including GLU305 and SER85 (pdP2X7 numeration) (Supporting material Figure 1).

During visual inspection (Table 1), compounds mimicking the conformation of JNJ47965567 (**5**, **6**, **7**, **8**, **9**, **10**, and **11**) and its interactions with ALA91, ASP92, TYR295, and LYS297 were selected for *in vitro* testing (Supporting material Table 1). Compound **4** was chosen as it interacts with SER86 (hP2X7 numbering) at the entrance of the pocket (similar to AZ10606120 and GW791343), while **2**, **4**, and **7** were chosen because they explored a novel and deeper region of the binding cleft (Figure 1C).

**Table 1:**
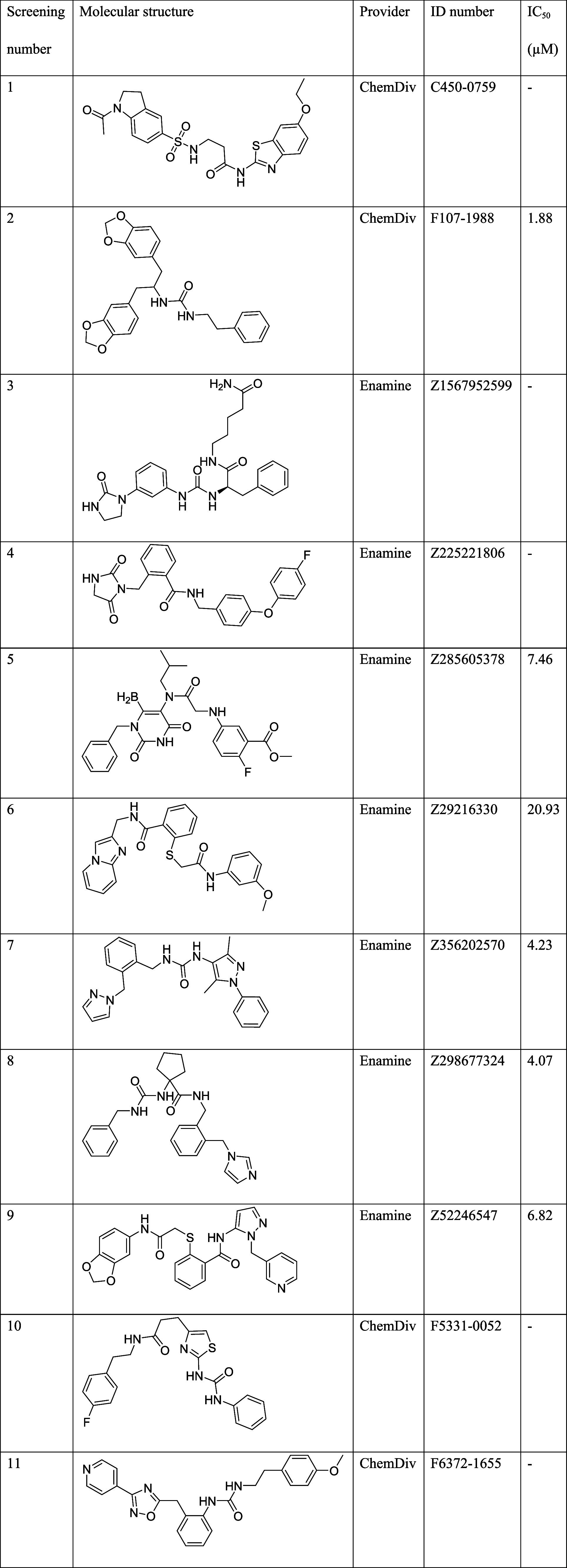
Compounds Selected from the VS Performed in the hP2X7 Negative Allosteric Pocket

Interestingly, JNJ47965567, the reference ligand, was also included in our screening hits, presenting a conformation very similar to the one cocrystallized in pdP2X7 and ranking among the top-scoring entries (Figure 1D). This suggested that the homology model and the VS method we used was a suitable technique for identifying hP2X7 modulators binding to this pocket. We did not see any other known P2X7 antagonists in the final pool of compounds selected from the virtual screen (8,194 entries).

### Initial Biological Evaluation Using YO-PRO 1 Dye Uptake Identifies 6 Potential Hit Candidates

The P2X7 receptor, besides being a ligand-gated ion channel, forms large membrane pores following prolonged exposure to high concentrations of ATP, leading to apoptosis (Mackenzie et al. 2005). The YO-PRO 1 dye uptake assay, which measures dye influx through these pores, was selected as the primary screen. The fluorescence of YO-PRO 1 increases when it binds to DNA, enabling real-time monitoring of P2X7 activation (calculated as the rate of increase in fluorescence as YO-PRO 1 binds nuclear DNA).^2^

To establish assay conditions, the EC_50_ of ATP on 1321 N1 cells stably transfected with hP2X7 was determined to be 697.4 ± 202.7 μM (Supporting material Figure 4A), using ATP concentrations ranging from 1 μM to 10 mM.

The screening compounds were tested at 30 μM (0.3% DMSO) in 5 μM YO-PRO 1 dye diluted in freshly prepared ECS-LD buffer. Cells pretreated with the screening compounds were challenged with 700 μM ATP, and the fluorescence intensities were recorded. The negative control consisted of cells treated with 0.3% DMSO, while the positive control was 0.1 μM JNJ47965567, a selective P2X7 antagonist.^45,46^

From the YO-PRO 1 dye uptake assay, six compounds—**2**, **5**, **6**, **7**, **8**, and **9**—statistically reduced P2X7 activation by at least 50% at 30 μM (Figure 2A).

**Figure 2:**
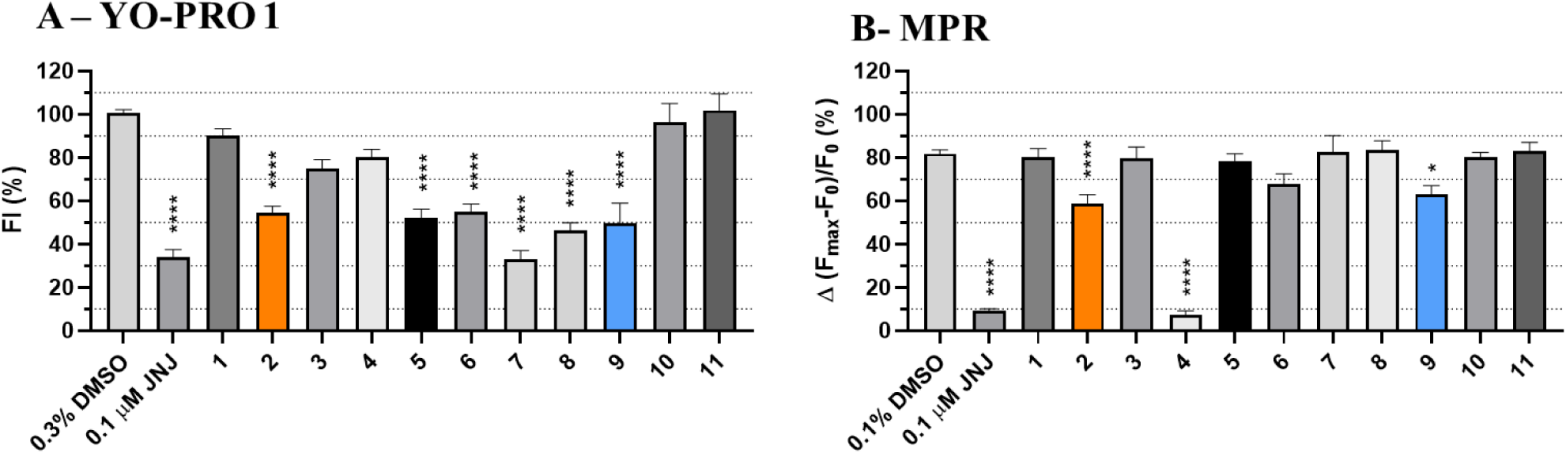
Initial screening assays to identify hit compounds. (A) The compounds were screened at 30 μM in the YO-PRO 1 dye uptake using 1321 N1 cells stably transfected with hP2X7. Compound activity was evaluated along a time of 20 min (75 s per cycle) after ATP addition (700 μM). The fluorescence intensity (FI) values of the first 10 cycles were considered for assessing their ability to inhibit hP2X7. Compound responses were normalized to the fluorescent signal recorded on 0.3% DMSO cells treated with 700 μM ATP of each plate. The graph shows the results obtained from three independent repeats (*n* = 4 per experiment). (B) Compounds screened at 10 μM using MPR using 1321 N1 cells stably transfected with hP2X7. The compound activity was evaluated for 5 min after ATP addition (15 s per cycle). The fluorescence intensity recorded in the first cycles (before reaching a plateau response) was considered for assessing the small molecule’s ability to inhibit hP2X7. Compound responses were normalized to the fluorescence signal recorded on 0.1% DMSO cells treated with 700 μM ATP of each plate. The graph shows the results obtained from three biological replicates (6 technical replicates per experiment; error bars represent the SEM from the means of each biological replicate (*n* = 3)). One-way ANOVA and Dunnet test were used for statistical analysis (****P < 0.0001), (*P < 0.02).

### Secondary Biological Assay Using Membrane Potential Red (MPR) Assay Confirms the Antagonist Activity of Compounds **2** and **9**

A secondary assay measuring changes in membrane potential was employed to confirm the hP2X7 inhibition. This assay utilizes a fluorescent dye that integrates into the membrane and fluoresces upon depolarization caused by the influx of ions caused by the activation of ion channels.^47,48^ The ATP EC_50_ calculated for 1321 N1 cells stably transfected with hP2X7 using the red version of the dye was 700 μM, very similar to the value recorded in the YO-PRO 1 dye uptake assay (Supporting material Figure 4B).

The screening compounds were tested at 10 μM in an ECS-LD buffer containing 1:10 diluted red dye (Figure 2B). Among the compounds tested, compounds **2**, **4**, and **9** statistically inhibited hP2X7-induced membrane depolarization. Although compound 4 gave rise to approximately 90% inhibition of P2X7 activity in the MPR assay, it showed no significant activity in the YO-PRO 1 dye uptake assay, potentially suggesting interference with the assay or a nonspecific mode of action. We decided to proceed to further characterize compounds **2** and **9** as they possessed P2X7 inhibitory activity in both assays.

### IC_50_ Determination of Compounds **2** and **9**

Compounds **2** and **9**, identified as hits, were further evaluated for concentration-dependent inhibition (IC_50_) of hP2X7 activation in the YO-PRO 1 dye uptake assay using HEK-293 cells stably transfected with hP2X7 (unfortunately, at this point in the study, the stably transfected 1321N1 cells lost P2X7 expression, and we were unable to recover them from frozen aliquots), for which the ATP EC_50_ was determined to be approximately 1 mM (Supporting material Figure 4C). Seven concentrations (100 to 0.03 μM) were tested, but due to precipitation at 100 μM, the DMSO concentration was increased to 1%.

Both compounds showed concentration-dependent inhibition of hP2X7 with IC_50_ values below 10 μM (**2**, IC_50_ = 1.875 μM; **9**, IC_50_ = 6.820 μM) (Figure 3A,B). These results confirmed that compounds **2** and **9** are good potential candidates for hit expansion and structure–activity relationship (SAR) studies.

**Figure 3:**
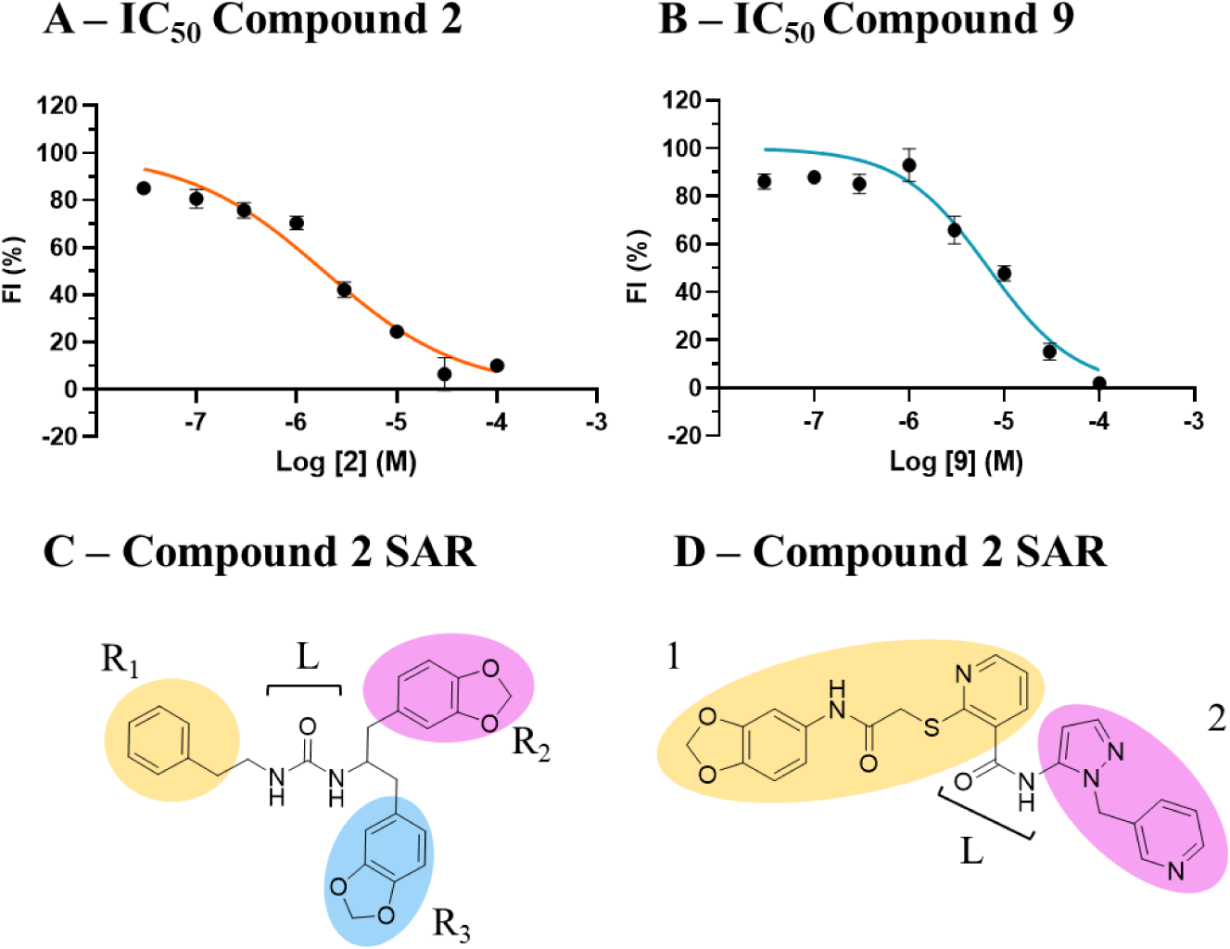
Inhibitory concentration response evaluation of compounds **2** and **9** and their groups highlighted for SAR. IC_50_ was determined for **2** (IC_50_ = 1.875 μM) (A) and **9** (IC_50_ = 6.820 μM) (B) by YO-PRO 1 (5 μM) assay. Compound activity was evaluated along a time of 20 min (75 s per cycle) after ATP addition (700 μM). The fluorescence intensity (FI) values of the first 10 cycles were considered for assessing their ability to inhibit hP2X7. The curves represent three biological replicates (*n* = 4 per replicate). Data were normalized by setting as 100% the FI recorded by hP2X7 HEK-293 cells incubated in 1% DMSO and stimulated by ATP, and as 0% the FI recorded by the same cells but treated with ECS-LD (assay buffer). (C) Highlighted areas of compound **2** considered for SAR study. R1 refers to the ethyl-benzyl group of the molecules, and R2 and R3 refer to the benzo-(1,3)-dioxol groups. (D) Highlighted areas of compound **9** considered for SAR study. We have defined the area highlighted in yellow and numbered as 1 as the “front” region of the molecule and the one colored in pink and named as 2 as the “back” region of the molecule. L highlights the urea and amide linkers of compounds **2** and **9**, respectively.

### Hit Expansion of Compound **2** and **9**

Structural analogues of compound **2** were identified using eMolecules, an online database of commercially available screening compounds (https://search.emolecules.com). The search of analogues was performed using the full structure of compound **2** as a query, as well as portions of the molecule, to discern the regions critical for target binding. Analogues with at least 70% structural similarity to compound **2** were selected for evaluation.

No small molecules with an identical linker length (*n* = 2) between the phenyl and the urea groups were available. Consequently, the ethylbenzyl moiety was named R1, and the two benzo­(1,3)-dioxol groups were named R2 and R3, as illustrated in Figure 3C.

Modifications in R1 explored different substitutions on the benzene ring (e.g., fluorine, ethoxy, and acetyl groups) or replacement with alkyl chains (**2a** and **2d**). The role of the urea linker was assessed using a single molecule in which it was substituted with an amide group (**2k**).

R2 remained unchanged in most analogues, except in **2j** and **2g,** where it was replaced by either 1,2-dimethoxybenzene (**2j**) or *p*-fluorobenzene (**2g**). Substitutions at R3 included *p*-fluorobenzene (**2d, 2g** and **2h**), and *p*-methoxybenzene (**2b, 2e,** and **2k**). In two analogues (**2f** and **2j**), R3 was removed entirely, resulting in a linear structure (Table 2).

**Table 2:**
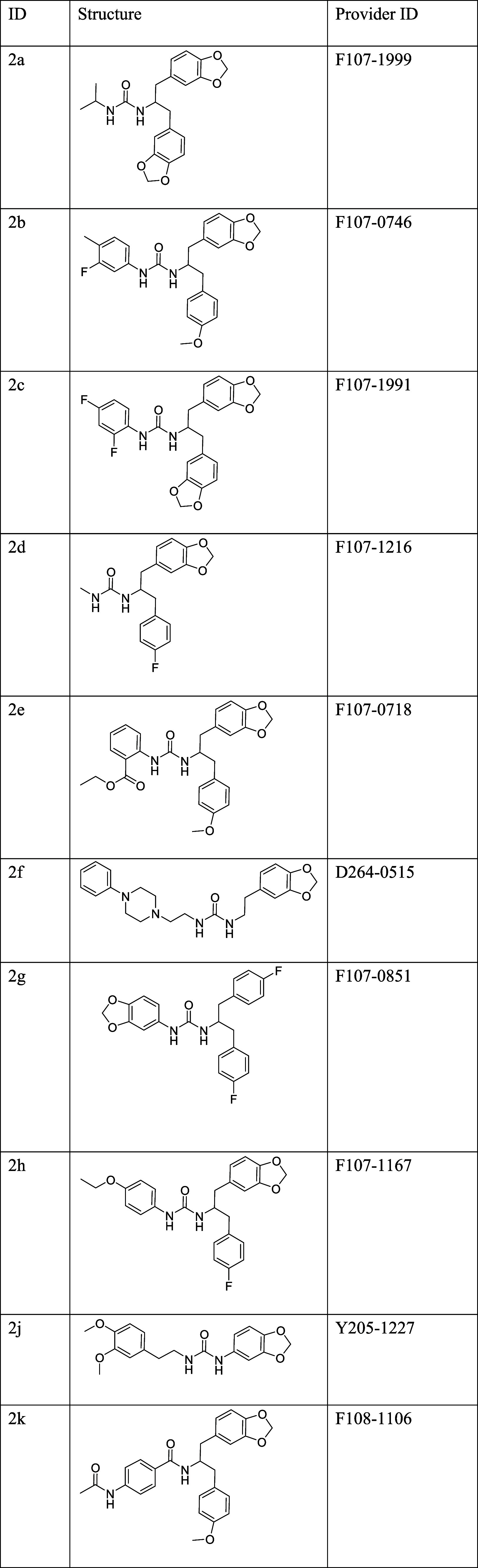
Commercially Available Structural Analogues of Compound **2** Purchased for SAR Study

The structural analogues were tested at 10 μM first in the YO-PRO 1 dye uptake assay (Figure 4A), with those reducing hP2X7 activity by 80% (**2c**, **2g**, **2h**, and **2k**) further evaluated in the MPR assay (Figure 4B).

**Figure 4:**
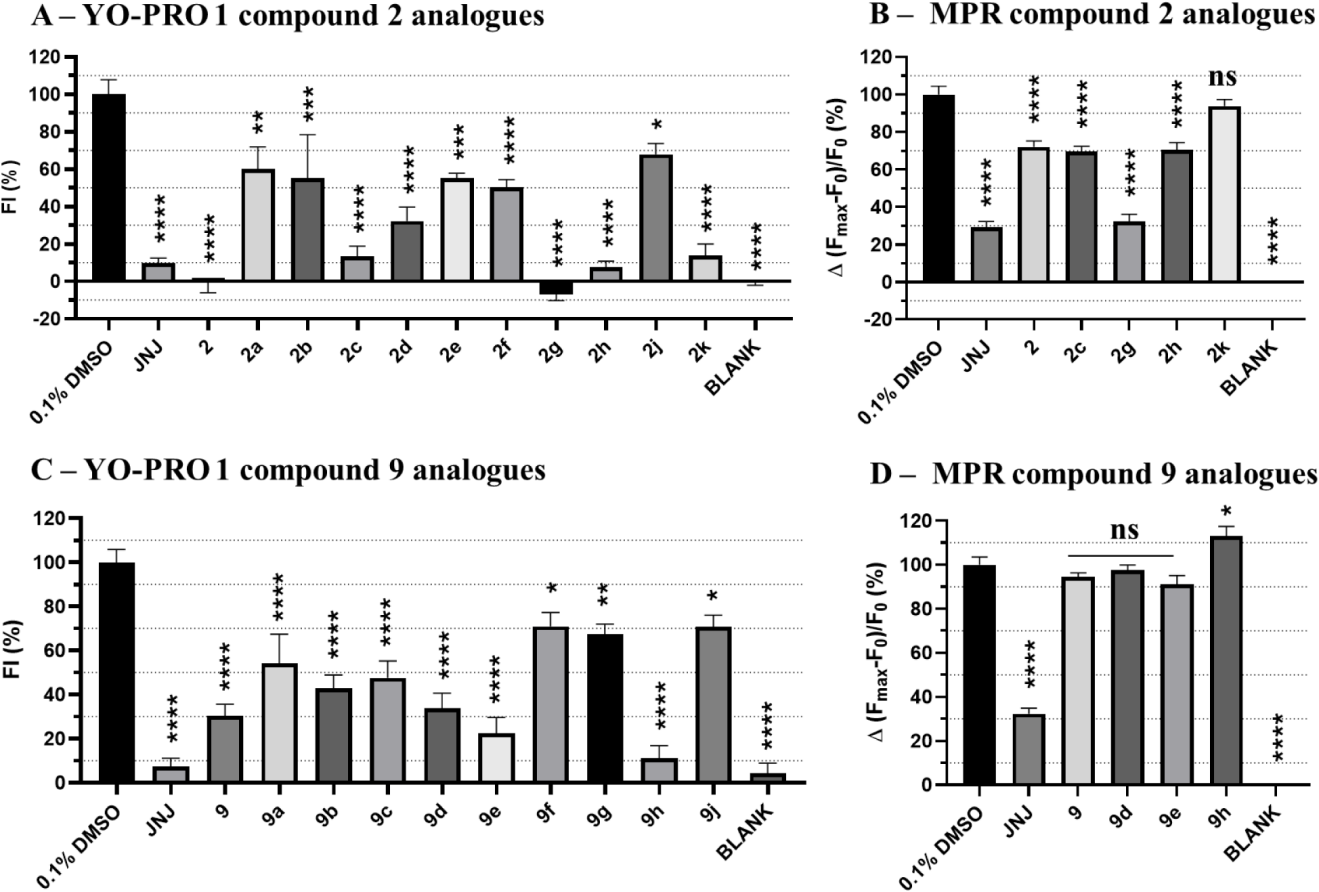
Evaluation of compound **2** and **9** analogue activity using YO-PRO 1 and MPR assays. The activity of compound **2** (A and B) and **9** (C and D) analogues was evaluated in YO-PRO 1 (A and C) and MPR (B and D) assays. The compounds were tested at 10 μM and preincubated for either 20 (YO-PRO 1) or 30 min (MPR). The baseline was recorded for 5 cycles before ATP treatment (1 mM). Fluorescence was recorded for 25 (YO-PRO 1) or 5 min (MPR). The data were normalized by setting as 100% for the fluorescence measured from hP2X7 HEK-293 cells incubated with vehicle (0.1% DMSO) and treated with 1 mM ATP and 0% for the fluorescence recorded by the cells not treated with ATP. The data shown correspond to three independent biological replicates (3 technical replicates per experiment; error bars represent the SEM from the mean values of each biological replicate (*n* = 3)). The statistical analysis was performed with one-way Anova using Dunnett’s function (P**** < 0.0001; P*** < 0.0003; P** < 0.002; P* < 0.02).

Among the compounds tested in the MPR assay, **2g** was the most potent compound that was able to inhibit hP2X7 by 70% (Figure 4 B).

Although a limited number of compounds were tested, results from the YO-PRO 1 dye uptake assay allowed for basic SAR analysis. The presence of an aromatic group in R1 was crucial for compound activity, as **2a** and **2d**, bearing either isopropyl or methyl groups at this position, showed reduced antagonist effects.

While R2 was mostly unvaried, its substitution with p-fluorobenzene (**2g**) retained potency comparable to that of the hit compound. The same modification in R3 enhanced activity, as observed for **2d** (compared to **2a**), **2g**, and **2h**. Conversely, the presence of p-methoxybenzene at R3 diminished hP2X7 inhibition, as seen for **2b** and **2e**, though **2k** still reduced receptor activation. The activity of **2k** may be attributed to either its R1 substitution or the replacement of the urea linker with an amide group.

Notably, the overall activity of this compound series appears to depend on its “Y” shape as the two linear molecules tested, **2f** and **2j**, exhibited reduced activity compared to **2**.

MPR assay data further suggest that the urea linker plays a critical role in activity as **2k** demonstrated diminished activity relative to the other structural analogues. Moreover, a double substitution with p-fluorobenzene at R2 and R3 (**2g**) increased compound activity in comparison to the molecule bearing just one at R3 (**2h**) or two 1,3-benzodioxole groups (**2** and **2c**).

Structural analogues of compound **9** were identified using the eMolecules database, employing fragments of its structure—front (1) and back (2) regions—as search queries (Figure 3D). The structures of the commercially available compounds are given in Table 3.

**Table 3:**
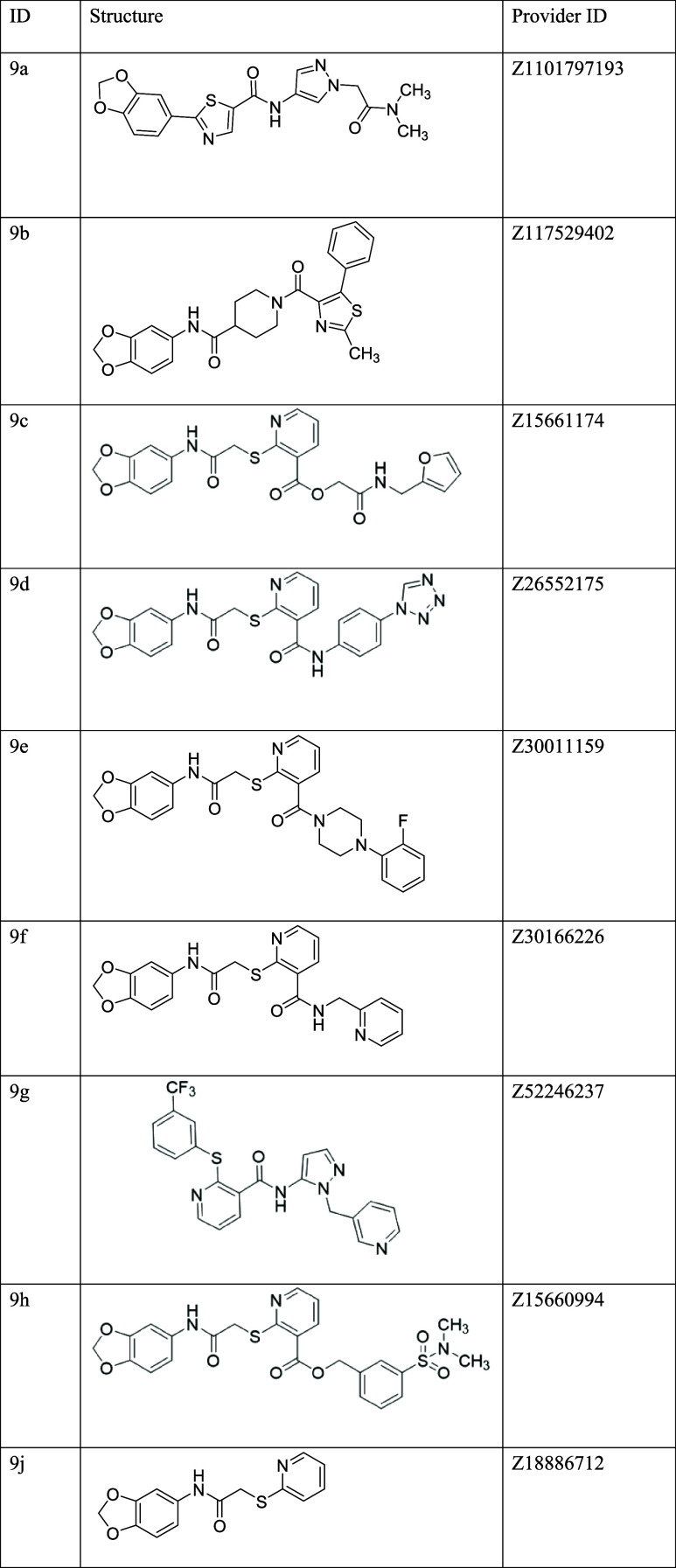
Commercially Available Structural Analogues of Compound **9** Purchased for SAR Study

Most of the selected analogues retained the front of compound **9** unchanged (Table 3) (**9c**, **9d**, **9e**, **9f**, **9g**, **9h**, and **9j**). The role of the amide connecting the front and back of compound **9** was explored in five molecules: it was preserved as a secondary amide in **9d** and **9f**, modified into a tertiary amide alkyl cycle in **9e**, or replaced with an ester group in **9c** and **9h**. Among the molecules available in eMolecules, only **9g** maintained the back region of **9** unvaried, making it a key candidate for assessing its contribution to activity. Additionally, **9a** (containing only the 1,3-benzodioxole) and **9b** (retaining both the 1,3-benzodioxole and the amide linker) were tested.

Structural analogues of compound **9** were initially screened at 10 μM using YO-PRO 1 dye uptake, with the most active compounds advancing to the MPR assay.

In the YO-PRO 1 dye uptake assay (Figure 4C), all analogues exhibited varying degrees of hP2X7 inhibition. However, only those with activity comparable to that of **9** (**9d**, **9e**, and **9h**) were further evaluated in the MPR assay. In this secondary screening, neither compound **9** nor its analogues significantly affected the membrane depolarization upon hP2X7 activation (Figure 4D).

The lack of inhibition of P2X7 observed with compound **9** was somewhat surprising, given that statistically significant inhibition (65% of control) was observed in the initial MPR screen (Figure 2B), but given this result, we focused on data from the YO-PRO 1 dye uptake assay to provide insights into compound **9** SAR. Notably, its activity was not strongly dependent on either the front (region 1) or back (region 2) (Figure 3D) of the molecule as both **9j** and **9g** exhibited a similar loss of activity toward P2X7. The importance of the amide adjacent to the 1,3-benzodioxol moiety was highlighted by the reduced activity of **9a** compared to that of **9b**.

Replacing the amide linker between the front and back regions with either an ester (**9h)** or a piperazine (**9e**) enhanced target inhibition, whereas extending the ester linker (**9c**) compromised activity. Additionally, maintaining direct linker attachment to an aromatic group was crucial for hP2X7 inhibition, as observed for **9d** but not for **9f**. Overall, replacing the amide linker with a piperazine adjacent to a p-fluorobenzene (**9e**) or an acetate (**9h**) linked to a sulfonamide-benzene enhanced the inhibitory effect of this family (Figure 4C).

### Compound **2g** IC_50_

Among the structural analogues of **2** and **9**, **2g** was identified as the most potent compound, showing activity in both the YO-PRO 1 dye uptake and MPR assays. Although we could only obtain this compound at >75% purity, its IC_50_ was calculated using the YO-PRO 1 dye uptake assay as 1.31 ± 0.2 μM, similar to that of compound **2** (1.875 μM) (Figure 5A).

**Figure 5:**
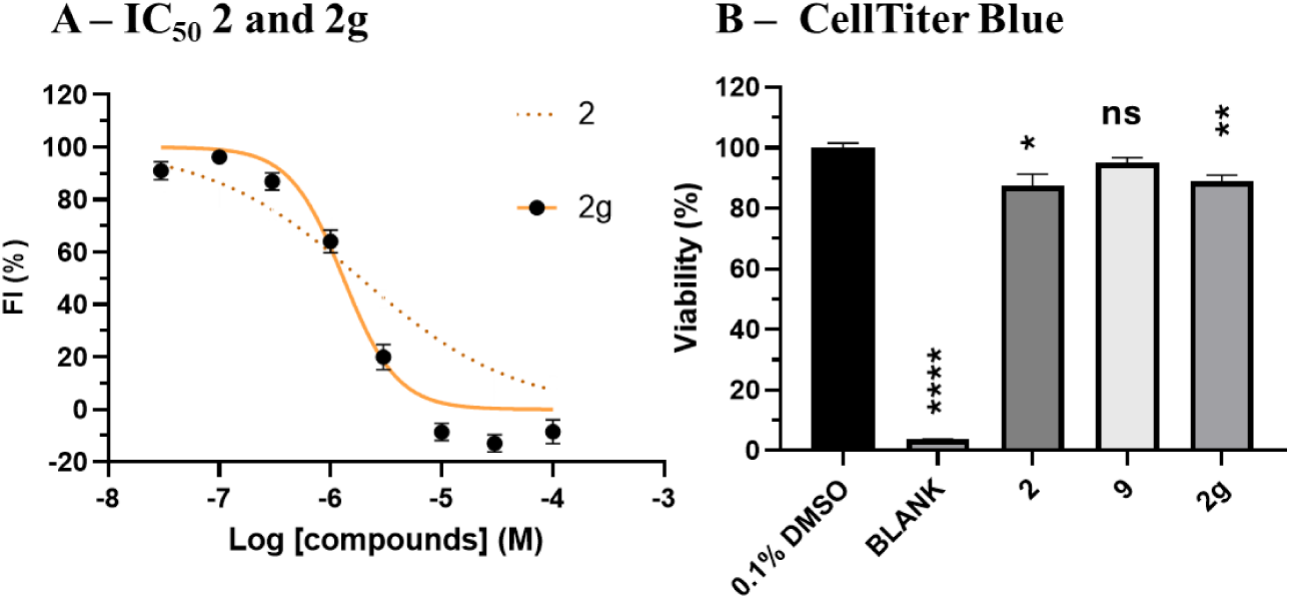
Concentration inhibition curve determination for compound **2g** (A) and evaluation of cytotoxicity for compounds **2**, **9**, and **2g**. (A) IC_50_ comparison between 2g (solid line) (1.31 ± 0.2 μM) and 2 (dotted line—data from Figure 3A) (1.875 μM). The data on the graph represent the mean from three independent experiments (*n* = 3 replicates per experiment). (B) The cytotoxicity of the compounds was evaluated on nontransfected HEK-293 cells using a CellTiter Blue assay. The cells were incubated for 24 h with the compounds at 10 μM. The fluorescent signal obtained from vehicle-treated cells (0.1% DMSO) was used to normalize the data to 100%, and the one obtained from wells not containing cells (BLANK) was set as 0%. The data are the combination of three biological replicates (error bars represent the SEM (*n* = 3)) analyzed by one-way ANOVA using Dunnet’s function (P**** < 0.0001; **< 0.002; * < 0.02; ns, non significant).

### Cell Viability Assay Using HEK-293 Cells

The potential cytotoxicity of the hit compounds identified, **2, 9**, and **2g**, was assessed at 10 μM by CellTiter Blue assay using nontransfected HEK-293 cells (Figure 5B).

After 24 h of treatment, no significant reduction in cell viability was observed for **9**. However, compounds **2** and **2g** slightly affected cell viability (∼10%), indicating minimal cytotoxicity at the tested concentration.

### Docking of Compound **2g** into the Negative Allosteric Pocket

To analyze the binding mode of compound **2g**, docking studies were conducted within the negative allosteric pocket, comparing its pose to those of compound **2** and the reference ligand JNJ47965567 (Figure 6).

**Figure 6:**
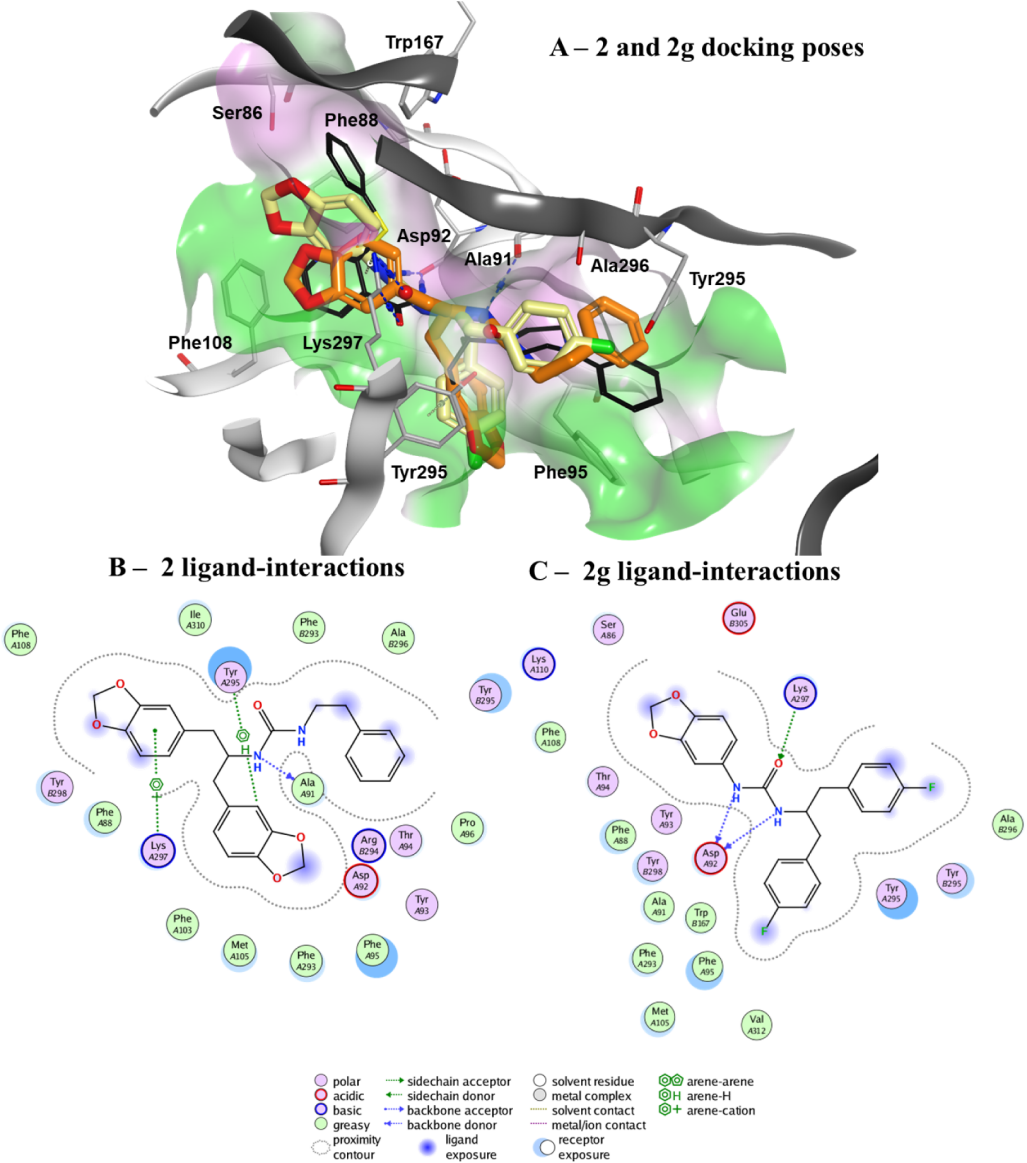
Predicted binding mode of **2g** to the hP2X7 negative allosteric pocket. (A) **2g** (carbon atoms in yellow), is overlaid with **2**, (carbon atoms in orange), and the cocrystallized JNJ47965567 (JNJ; black sticks). The receptor binding surface is represented in green (lipophilic) and pink (hydrophilic). Interactions between the ligands, **2** and **2g**, and the receptor are represented in blue (hydrogen bonds) and green dotted lines (lipophilic interactions). (B) Compound **2** ligand interaction diagram. (C) Compound **2g** ligand interaction diagram (see key above).

Both compounds **2** and **2g** adopted a “Y”-shaped conformation within the pocket, reminiscent of JNJ47965567. Specifically, the p-fluorobenzene groups of **2g**, and ethylbenzene and benzo-(1,3)-dioxol groups of **2**, aligned with the bent conformation of JNJ47965567, corresponding to its tetrahydro-4-pyranyl-4-phenylpiperazine. However, compounds **2** and **2g** extended further into the lipophilic region of the binding site. Compound **2** formed hydrogen bonds with the backbone of ASP92, a hydrogen-arene interaction with TYR295, and a cation-arene interaction with LYS297. In contrast, **2g** established three hydrogen bonds: two with the backbone of ASP92 and one with the side chain of LYS297. Despite the lack of explicit lipophilic interactions in the ligand-interaction diagram, the p-fluorobenzene group of **2g** likely interacts with TYR295 in chain A of hP2X7 in the same manner as the benzo-(1,3)-dioxol group of **2**, as suggested by their spatial overlap (Figure 6).

## Discussion

In this work, computational methods, including 3D pharmacophore modeling and VS targeting the negative allosteric pocket of an hP2X7 homology model, led to the identification of three small molecules with inhibitory activity in the low micromolar range.

Previous VS studies have primarily focused on the ATP pocket,^30,31^ with one targeting the negative allosteric pocket.^38^ Among these, two studies created human P2X7 homology models derived from zebrafish P2X4 ^30^ and pdP2X7 ^31^ (5U2H). Ferreira and colleagues, instead, screened their databases against the pdP2X7 structure in complex with JNJ47965567,^38^ the same structure used as a template in our study. Of these, the pdP2X7 structure can be considered the most suitable as it presents higher resolution (3.2 Å for 5U1X, 3.9 Å for 5U2H) and greater sequence identity to hP2X7 than the ones considered by Caseley and Zhao (84.5% for giant panda P2X7 and 47.8% for zebrafish P2X4).^30,31^ However, future VS efforts may benefit from the recently resolved rat P2X7 cryo-EM structures in complex with negative allosteric antagonists, which present higher resolution (2.18–2.78 Å) and an improved visualization of key protein–ligand interactions.^26^

To refine the screening process, a ligand-based approach was employed prior to VS. A 3D pharmacophore model was developed based on five chemical features shared by known P2X7 negative allosteric modulators (A740003, A804598, and JNJ47965567). Similarly to our ligand-based approach, Ferreira and colleagues focused on filtering natural product databases, considering the shape of JNJ47965567.^38^ This approach facilitated the identification of structurally novel inhibitors. Indeed, structural similarity analysis using MACCS (Molecular ACCess System) fingerprints and MOE clustering confirmed that none of the 11 selected compounds shared >60% similarity (Tanimoto) with 60 known P2X7 antagonists, underscoring the novelty of our hits (Supporting material Figure 2).

Key ligand-target interactions of five cocrystallized antagonists to P2X7 were analyzed through MOE ligand interaction diagrams before visually inspecting the small molecules resulting from our VS. Contrary to Karasawa and Kawate’s observations, we noticed that not all the antagonists interacted with lipophilic residues (PHE88, PHE95, MET105, PHE108, TYR295, and ILE310).^25^ Instead, we observed some hydrogen bonding with SER85, ASP92, LYS210, LYS297, and GLU305 (pdP2X7 numbering), which were not mentioned previously (Supporting material, Table 1). In contrast, Ferreira’s group detected most of the lipophilic interactions highlighted by Kawate, in addition to hydrogen bonding to TYR298 and ASP92.^25,38^ The recently published antagonist-bound rat P2X7 cryo-EM structures further support our findings, revealing hydrogen bonds with ASP92 and LYS297, which were previously undetected by Kawate.^25,26^

During compound selection, molecules exhibiting conformational similarity to the cocrystallized JNJ47965567 and key interactions with critical residues were prioritized, following an approach similar to Ferreira et al.^38^ The activity of the compounds selected on the VS was evaluated using YO-PRO 1 dye uptake and MPR assays, assessing ATP-stimulated P2X7 macropore formation and ion channel opening, respectively. The use of two or more complementary assays for assessing P2X7 modulator activity has been previously applied to help identify false positives^48^ as macropore formation is also linked to P2X2 and P2X4.^9,49^ As P2X receptors are ion channels, they are probably best studied using electrophysiology techniques such as single-cell patch clamp, but these techniques can be laborious when testing libraries of compounds.^50^ For this reason, we used the MPR assay, a plate-format membrane potential assay (Molecular Devices) previously used by other research groups.^47,48^ During our assay optimization, the red dye gave an ATP EC_50_ (678.9 μM; Supporting material Figure 4) comparable to the one calculated in the YO-PRO 1 dye uptake assay (697.4 μM), which is why we decided to use the red dye in our experiments rather than the blue dye (which has previously been used in compound screening^27,47^ but, in our experiments, gave an EC_50_ of 2,052 μM). Of the 11 tested compounds, six (**2**, **5**, **6**, **7**, 8, and **9**) exhibited antagonist activity in the YO-PRO 1 assay, while only two of those (**2** and **9**) were active in the MPR assay, likely due to lower testing concentrations or indirect inhibition mechanisms. It is important to note that P2X7-dependent macropore formation has previously been attributed to the activation of secondary channels, including pannexin-1 ^10^ and so we cannot rule out the possibility that the compounds blocking YO-PRO 1 uptake, which did not also block in the MPR assay (**5**, **6**, **7**, and **8**), may be acting indirectly to block the macropore but not the channel.

Compounds **2** and **9** were further characterized through inhibitory concentration–response assays and by profiling their SAR using commercially available structural analogues of the hit compounds.^32,51^ While convenient, this approach does not permit the systematic SAR study performed by synthesizing *de novo* structural analogues bearing a single modification at the time as it depends on the analogues available for purchase. Nevertheless, we were able to select a small series of structural analogues, enabling us to analyze a limited SAR of compounds **2** and **9**.

Compound **2** is characterized by a “Y” shape, where 3 “R” groups are arranged around a urea linker. Its activity was substantially reduced by the removal of R3 (**2f, 2j**), emphasizing the importance of a lipophilic group in this region. An aromatic group in R1 was beneficial, though substituent effects require further study. The replacement of the urea linker with an amide (**2k**) reduced the activity. Overall, the presence of a p-fluorobenzene in R3, as in **2d**, **2g**, and **2h**, enhanced the potency.

Compound **9** displayed activity lower than that of **2**, as suggested by its IC_50_. SAR analysis focused on three molecular regions: the front, the back, and the amide linker. Analogues **9g** and **9j**, lacking either the back or the front regions of **9,** respectively, were among the least active molecules, highlighting the importance of functional groups in these two areas. Limited analogue availability prevented a comprehensive study of the back region. Among the structural analogues sharing the front of compound **9** (**9j**, **9c**, **9d**, **9e**, and **9h**) **9d**, **9e**, and **9h** were the most active. Notably, these three molecules featured different linkers: amide (**9d**), cyclic amide (**9e**), and ester group (**9h**), suggesting that amides are not strictly necessary for activity.

Besides having briefly studied compound **2** and **9** SAR, we identified **2g** (obtained from the vendor at >75% purity) as having similar potency to **2**. Comparing docking poses of compounds **2** and **2g** in our model of hP2X7 revealed a similar binding mode, with **2g** forming additional hydrogen bonds with ASP92 and LYS297, potentially enhancing target affinity.

Overall, the identified compounds found in this work exhibit comparable activity to those discovered in previous P2X7 VS campaigns.^30–32^ It should be noted that while we used two distinct functional assays to screen for hit candidates (YO-PRO 1 dye uptake reporting large cation flux and MPR measuring changes in membrane potential), we did not directly measure P2X7 activity using patch-clamp electrophysiology or ATP-stimulated calcium influx, and further characterization of our hit compounds should also utilize these methods. Furthermore, our compounds displayed only modest inhibitory activity (in the low micromolar range), and it is important to note that significant further medicinal chemistry optimization is needed to develop these molecules into viable clinical candidates with nanomolar potency.

## Conclusions

This work employed a combination of LBDD and SBDD VS approaches to identify novel hP2X7 antagonists targeting the negative allosteric pocket. Two complementary functional assays, assessing distinct P2X7 features, were crucial in identifying three hit compounds: **2**, **2g**, and **9**. Among these, the compound **2** family exhibited higher inhibitory activity compared to **9**, making it a promising candidate for further optimization. Future medicinal chemistry efforts should focus on systematically modifying substituents at the three “R” positions. In particular, elongation of R1 may enhance interactions with the entrance of the negative pocket, as observed in AZ10606120 and GW791343. Additionally, the effect of various aromatic substitutions at R2 and R3 warrants further investigation.

## Supplementary Material





## Data Availability

All data used in this study are available freely. The pdb files used for virtual screening and those containing active compound dockings are provided freely at zenodo.org with DOI: 10.5281/zenodo.15145380.

## Notes

Small molecules used for pharmacophore search (Supporting Material Figure 1); small molecules considered for comparison of structural similarity with the compounds found in our VS (Supporting Material Figure 2); ligand–protein interaction diagrams of five known P2X7 antagonists (Supporting Material Figure 3); types of interactions between the 11 compounds chosen from the VS (Supporting Material Table 1); ATP EC_50_ values calculated in YO-PRO1 and MPR assay for cells stably transfected with human P2X7 (Supporting Material Figure 4). (PDF)
The “SMILE_screening_compounds.csv” file lists the SMILES ID for all the screening compounds, with the most potent compounds highlighted in yellow. The “Compound data” file contains vendor HPLC traces indicating compound purity and proof of purchase. (PDF)

## Abbreviations

AZ: AZ10606120
cryo-EM: cryoelectron microscopy
hP2 × 7: human P2X7
F: fluorescence
FI: fluorescence intensity
HTS: high-throughput screening
IC50: concentration-dependent inhibition
JNJ: JNJ47965567
LBDD: ligand-based drug design
MOE: Molecular Operating Environment
MPR: Membrane Potential Red assay
nfP2X7: nonfunctional P2X7
pdP2X7: giant panda P2X7
RMSD: root-mean-square deviation
SAR: structure–activity relationship
SBDD: structure-based drug design
SP: Standard Precision
VS: virtual screening
XP: Extra Precision
