# Supplementary material for: Identification of New Human P2X7 Antagonists Using Ligand- and Structure-Based Virtual Screening
Source: J Chem Inf Model. 2025 Jun 26;65(13):7143–55. doi: 10.1021/acs.jcim.5c00552 (PMC12264943; doi:10.1021/acs.jcim.5c00552)
Supplement: Supplementary file 1 [file ci5c00552_si_001.pdf]

Identification of new human P2X7 antagonists using ligand- and structure-based virtual screening

Marika Zuanon[1], *Andrea Brancale*[2], *Mark T. Young*[1]*

AUTHOR ADDRESS

[1]School of Biosciences, Sir Martin Evans Building, Cardiff University

[2] Department of Organic Chemistry, University of Chemistry and Technology, Prague

*** youngmt@cardiff.ac.uk**

Supporting Information

Number of Pages: 8
Number of Figures: 4
Number of Tables: 1

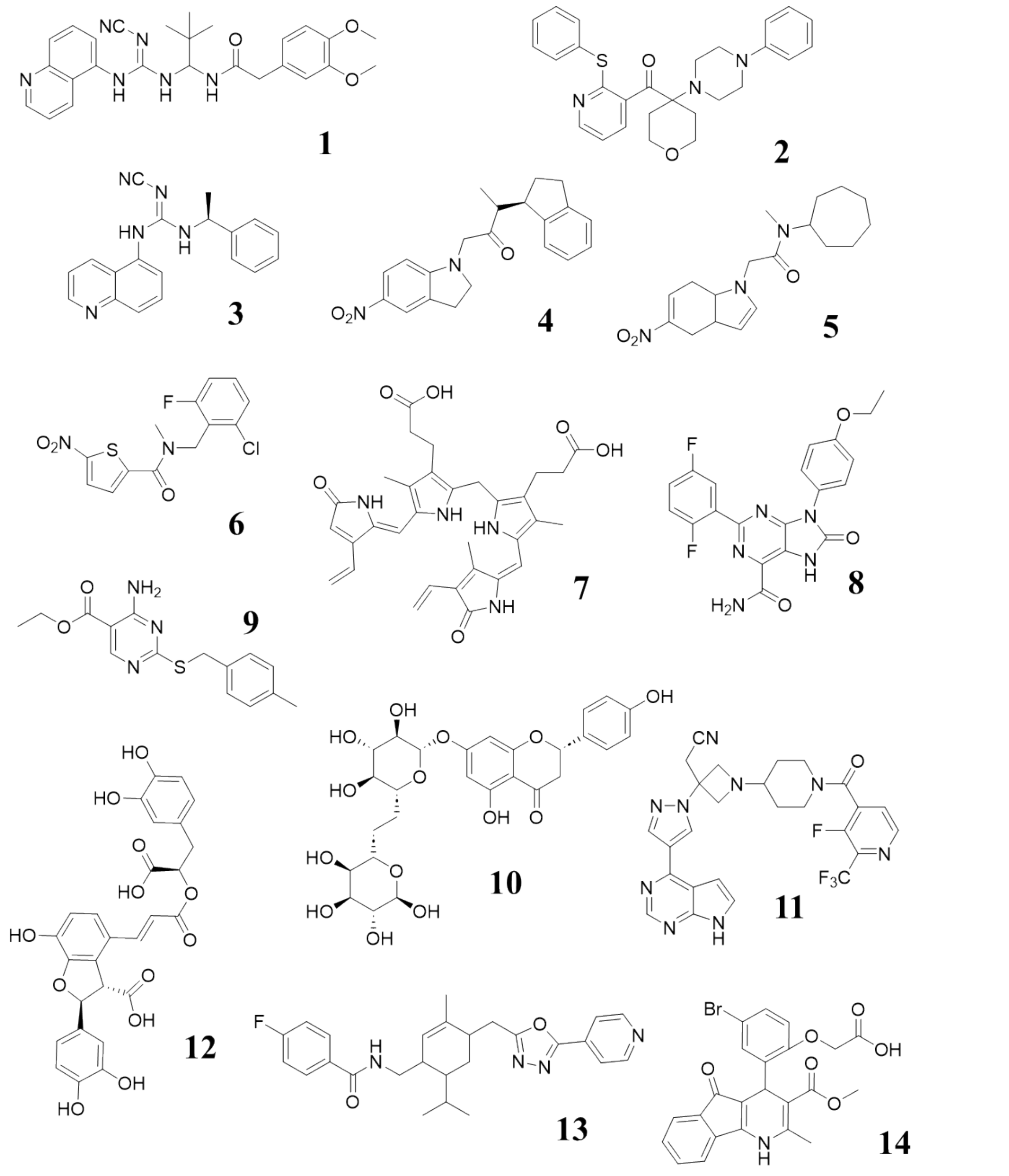

Supporting material Figure 1. P2X7 negative allosteric modulators used for Pharmacophore search and discovered by VS campaigns. 1-3 [1]; 4-6 [2], 7-12 [3], 13 [4] and 14 [5].

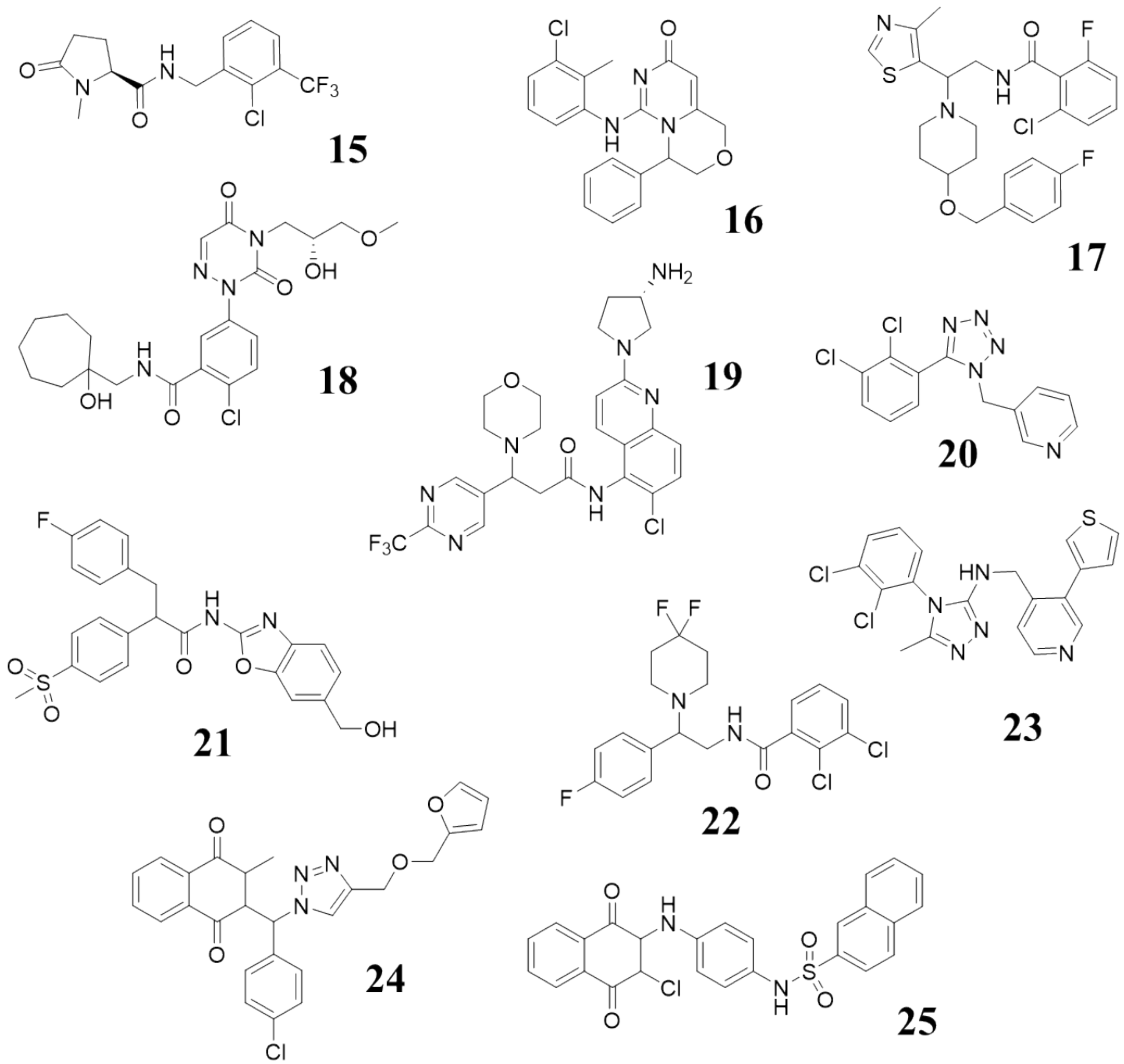

Supporting material Figure 2. Selected P2X7 negative allosteric modulators used for structure similarity comparison with the small molecules we selected in our VS. (15 [6]; 16-17-22 [7]; 18 [8]; 19 [9], 21 [10]; 20, 23 [11]; 24, 25 [12].

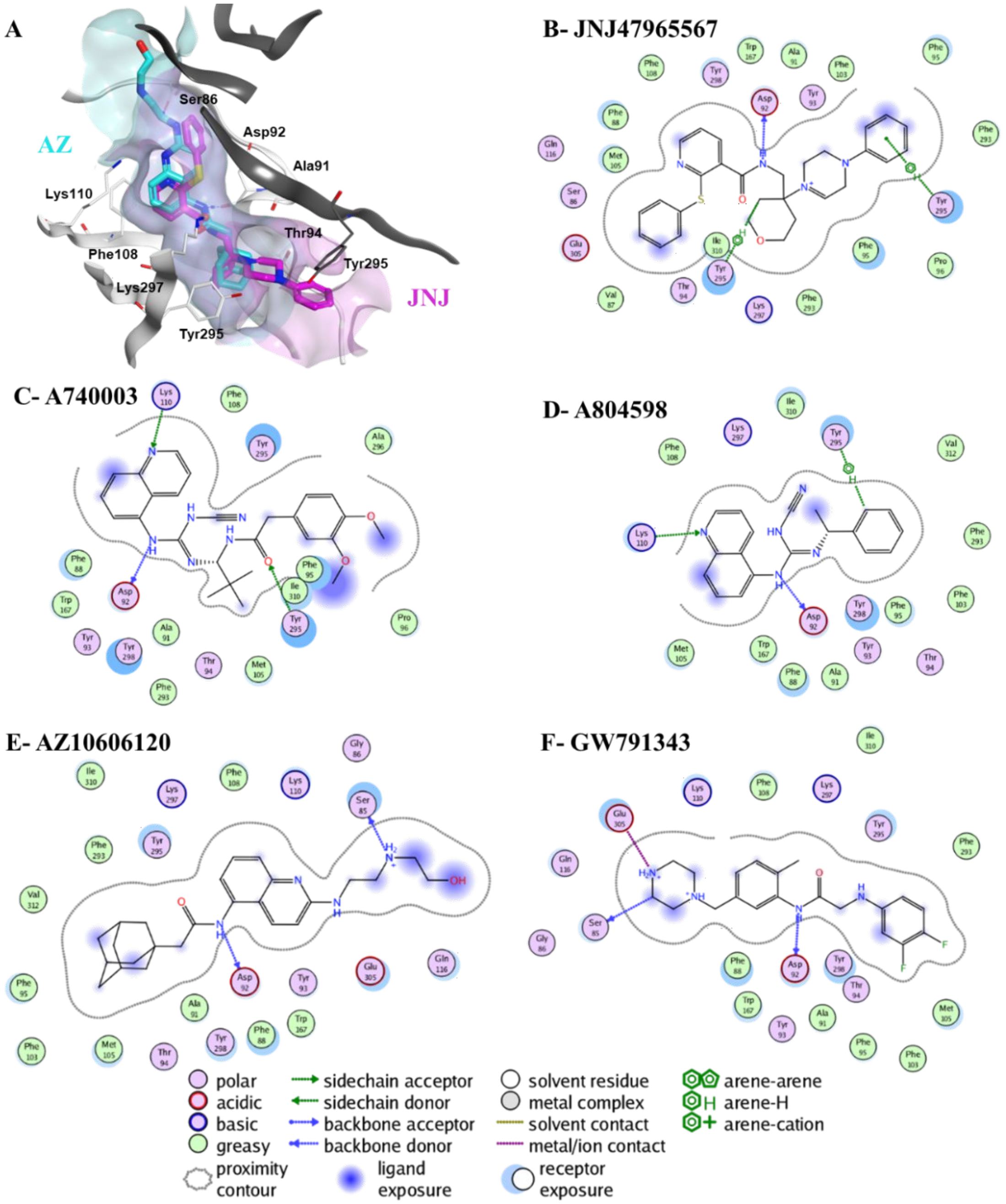

Supporting material Figure 3. Ligand interactions in the negative allosteric pocket. A) Superimposition of panda P2X7 crystal structures with bound JNJ47965567 (PDB:5U1X) (pink) and AZ10606120 (PDB: 5U1W) (cyan); JNJ= JNJ47965567; AZ= AZ10606120. The negative

allosteric pocket areas occupied by AZ10606120 and JNJ47965567 are highlighted in cyan and pink, respectively. P2X7 chains are colored in grey and black. B-F) Ligand-Protein interaction diagrams of 5U1X (JNJ47965567) (B), 5U1U (A740003) (C), 5U1V (A804598) (D), 5U1W (AZ10606120) (E), and 5U1Y (GW791343) (F). Amino acids colored in pink are polar residues, divided into acidic (red edge), and basic (blue edge). Amino acids colored in green are lipophilic residues. Sidechain and backbone interactions are represented by green and blue arrows, respectively (see key above).

Supporting material Table 1. Residues interacting with the screened compounds. The interactions between the compounds selected during visual inspection and the hP2X7 negative allosteric pocket were visualized using Ligand-interaction diagrams in MOE. ‘H(d)’ and ‘H(a)’ stand for hydrogen donor or acceptor bond, while ‘s’ and ‘b’ specify if the residues are involved in the interaction through their sidechain or backbone. ‘Ar-H’ and ‘Ar-Cat’ refer to an interaction Arene-hydrogen and Arene-Cation between ligand and protein.

Screening Number	SER85	PHE88	ALA91	ASP92	THR94	PHE95	PHE108	TRP167	TYR295	LYS297	ALA296
1		Ar-H	H (d)b			Ar-H	Ar-H		Ar-H	Ar-Cat	
2				H (d)b							
3		Ar-H	H (d)b	H (d)b	H (d)b			Ar-H	Ar-H	Ar-Cat	
4	H (d)s	Ar-H		H (d)b						H (d)s	
5					H (d)b				Ar-H	H (a) s<br>Ar-Cat	H(a)b
6		Ar-H		H (d)b	H (d)b				Ar-H	Ar-Cat	
7			H (d)b				Ar-H	Ar-H		Ar-Cat	
8		Ar-H	H (d)b	H (d)b					Ar-H	Ar-Cat	
9			H (d)b	H (d)b			Ar-H	Ar-H	Ar-H		
10		Ar-H	H (d)b		H (d)				Ar-H	H (a)s	
11			H (d)b	Ar-H					Ar-H	H (a)s	

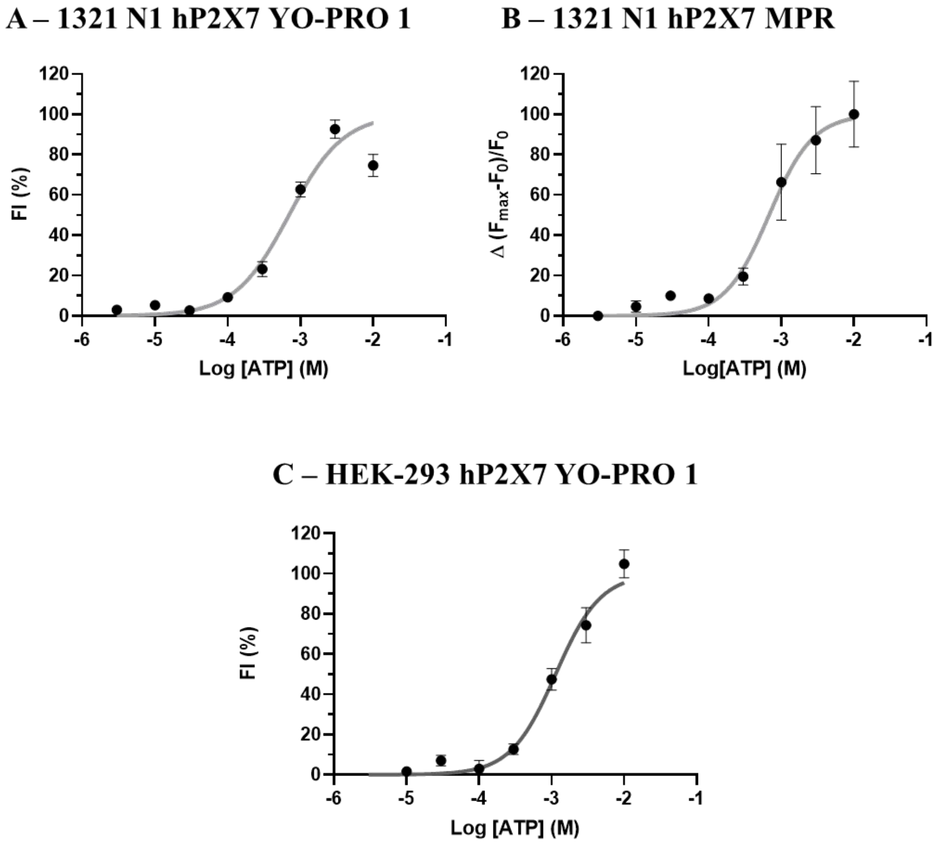

Supporting material Figure 4. ATP concentration-response curves for the human P2X7-expressing cell lines used in this study. A) The ATP concentration-response curve was determined in 1321 N1 hP2X7 cells by calculating the gradient of YO-PRO 1 uptake 10 cycles (25 min) after ATP addition. The ATP EC_{50} calculated from three biological replicates (6 technical replicates per experiment; error bars represent the SEM from the means of each biological replicate (n=3)) was 697.4 ± 202.7 μM, Hill Slope: 1.161. B) An ATP concentration-response curve (EC_{50}= 678.9 μM; one biological replicate, (6 technical replicates; error bars represent the SEM) was calculated in the MPR assay, after monitoring the response for 10 cycles (5 min) after ATP addition. C) An ATP concentration-response curve was determined in HEK-293 hP2X7 cells by calculating the

gradient of YO-PRO 1 uptake 10 cycles (25 min) after ATP addition. The ATP EC_{50} obtained from three biological replicates (6 technical replicates per experiment; error bars represent the SEM from the means of each biological replicate (n=3)) was 1138.00 ± 238.95 μM, Hill Slope: 1.397.

SUPPORTING REFERENCES

(1) Karasawa, A.; Kawate, T. Structural Basis for Subtype-Specific Inhibition of the P2X7 Receptor. *Elife* **2016**, *5* (DECEMBER2016), 1–17. https://doi.org/10.7554/eLife.22153.

(2) Caseley, E. A.; Muench, S. P.; Fishwick, C. W.; Jiang, L. H. Structure-Based Identification and Characterisation of Structurally Novel Human P2X7 Receptor Antagonists. *Biochem. Pharmacol.* **2016**, *116*, 130–139. https://doi.org/10.1016/j.bcp.2016.07.020.

(3) Zhao, Y.; Chen, X.; Lyu, S.; Ding, Z.; Wu, Y.; Gao, Y.; Du, J. Identification of Novel P2X7R Antagonists by Using Structure-Based Virtual Screening and Cell-Based Assays. *Chem. Biol. Drug Des.* **2021**, *98* (1), 192–205. https://doi.org/10.1111/cbdd.13867.

(4) Ferreira, N. C. da S.; Viviani, L. G.; Lima, L. M.; Amaral, A. T. do; Romano, J. V. P.; Fortunato, A. L.; Soares, R. F.; Alberto, A. V. P.; Coelho Neto, J. A.; Alves, L. A. A Hybrid Approach Combining Shape-Based and Docking Methods to Identify Novel Potential P2X7 Antagonists from Natural Product Databases. *Pharmaceuticals* **2024**, *17* (5). https://doi.org/10.3390/ph17050592

(5) Pasqualetto, G.; Zuanon, M.; Brancale, A.; Young, M. T. Identification of a Novel P2X7 Antagonist Using Structure-Based Virtual Screening. *Front. Pharmacol.* **2023**, *13* (January), 1–17. https://doi.org/10.3389/fphar.2022.1094607.

(6) Abdi, M. H.; Beswick, P. J.; Billinton, A.; Chambers, L. J.; Charlton, A.; Collins, S. D.; Collis, K. L.; Dean, D. K.; Fonfria, E.; Gleave, R. J.; Lejeune, C. L.; Livermore, D. G.; Medhurst, S. J.; Michel, A. D.; Moses, A. P.; Page, L.; Patel, S.; Roman, S. A.; Senger, S.;

Slingsby, B.; Steadman, J. G. A.; Stevens, A. J.; Walter, D. S. Discovery and Structure-Activity Relationships of a Series of Pyroglutamic Acid Amide Antagonists of the P2X7 Receptor. *Bioorganic Med. Chem. Lett.* **2010**, *20* (17), 5080–5084. https://doi.org/10.1016/j.bmcl.2010.07.033.

(7) Dane, C.; Stokes, L.; Jorgensen, W. T. P2X Receptor Antagonists and Their Potential as Therapeutics: A Patent Review (2010–2021). *Expert Opin. Ther. Pat.* **2022**, *32* (7), 769–790. https://doi.org/10.1080/13543776.2022.2069010.

(8) Duplantier, A. J.; Dombroski, M. A.; Subramanyam, C.; Beaulieu, A. M.; Chang, S. P.; Gabel, C. A.; Jordan, C.; Kalgutkar, A. S.; Kraus, K. G.; Labasi, J. M.; Mussari, C.; Perregaux, D. G.; Shepard, R.; Taylor, T. J.; Trevena, K. A.; Whitney-Pickett, C.; Yoon, K. Optimization of the Physicochemical and Pharmacokinetic Attributes in a 6-Azauracil Series of P2X 7 Receptor Antagonists Leading to the Discovery of the Clinical Candidate CE-224,535. *Bioorganic Med. Chem. Lett.* **2011**, *21* (12), 3708–3711. https://doi.org/10.1016/j.bmcl.2011.04.077.

(9) Hamburgen, W.; Whitted, W.; Clidaras, J.; Carlson, A.; Aigner, G.; Beaty, D. L. Patent Application Publication Pub . No .: US 2011 / 0008813 A1. **2011**, *1* (19).

(10) Brotherton-Pleiss, C. E.; Walker, K. A. M. Amido Compounds. US20120149718A1, 2012.

(11) Park, J. H.; Kim, Y. C. P2X7 Receptor Antagonists: A Patent Review (2010–2015). *Expert Opin. Ther. Pat.* **2017**, *27* (3), 257–267. https://doi.org/10.1080/13543776.2017.1246538.

(12) Furtado Pacheco, P. A.; Tadeu Gomes Gonzaga, D.; Lidmar von Ranke, N.; Rangel Rodrigues, C.; Rodrigues da Rocha, D.; de Carvalho da Silva, F.; Ferreira, V. F.; Faria, R. X. Studies of Naphthoquinone Sulfonamides and Sulfonate Ester. **2023**.
